# Precision Medicine and Artificial Intelligence: Beyond Obesity Care

**DOI:** 10.31662/jmaj.2026-0299

**Published:** 2026-07-31

**Authors:** Shigeki Matsubara

**Affiliations:** 1 Department of Obstetrics and Gynecology, Jichi Medical University, Tochigi, Japan; 2 Department of Obstetrics and Gynecology, Koga Red Cross Hospital, Koga, Japan; 3 Medical Examination Center, Ibaraki Western Medical Center, Chikusei, Japan

**Keywords:** artificial intelligence, obesity, precision medicine, privacy

## Dear Editors,

The narrative review by Bastías-Pérez ^(1)^ is highly significant. Although categorized as a narrative review, the article also offers the author’s thoughtful ethical perspective regarding the application of artificial intelligence (AI) to precision medicine in obesity. I found this feature particularly valuable. I agree with the author’s overall message.

Bastías-Pérez was concerned about privacy, transparency, accountability, equity, shared decision-making, and social justice. I was reminded that these concerns may not be specific to obesity care.

Having been engaged in obstetrics and gynecology (OBGYN) practice for 47 years, I have encountered most of these concerns in OBGYN practice as well. OBGYN practice routinely involves highly private and thus sensitive information, including reproductive history, genetic information, family history, behavioral factors, and highly complex individualized decision-making. In my practice, I have encountered several cases that raised concerns about privacy and shared decision-making. Some adolescent girls presented with genital hemorrhage and were ultimately diagnosed with ectopic pregnancy. Such information is greatly related to privacy and highly confidential. In another case, antenatal fetal ultrasound revealed some nearly fatal conditions, and then resuscitation at birth or neonatal surgery required highly complicated shared decision-making. The issue involves both the infant and parents ^(2)^. I believe similar observations may apply to many other medical specialties.

This perspective led me to consider whether the ethical concerns highlighted by the author arise primarily from obesity itself or from a broader convergence of precision medicine and AI. Precision medicine requires increasingly detailed personal information to tailor care to the individual. AI may further amplify both the utility and vulnerability of such data through large-scale collection, integration, and analysis. As for the above-mentioned OBGYN issues, such data accumulation and AI analysis may provide data for precision medicine on adolescent gynecology and fetal disorders. Thus, AI may not create entirely new ethical challenges but rather intensify existing ones.

If I, as an OBGYN specialist, had written about AI use in precision medicine, I would have naturally focused on OBGYN issues. The cases described above are examples. Thus, it is quite natural that Bastías-Pérez focused on obesity: obesity provides an important and highly relevant example. Also, he did not suggest that these concerns are unique to obesity care. However, emphasizing obesity may inadvertently lead some readers, especially less experienced readers, to regard these concerns as unique to obesity care. The issues discussed by the author may be generalizable to many fields in which precision medicine and AI intersect.

## Article Information

### Acknowledgments

ChatGPT-5 was used to assist with grammar checking.

### Author Contributions

Shigeki Matsubara conceived and wrote the manuscript.

### Conflicts of Interest

None
